# Women with maternal near-miss in the intensive care unit in Yangzhou, China: a 5-year retrospective study

**DOI:** 10.1186/s12884-021-04237-y

**Published:** 2021-11-19

**Authors:** Ying Chen, Jiaoyang Shi, Yuting Zhu, Xiang Kong, Yang Lu, Yanru Chu, Miskatul Mustafa Mishu

**Affiliations:** 1 School of Nursing, Yangzhou University, Yangzhou, Jiangsu Province China; 2 Department of Obstetrics, The Eighth Affiliated Hospital of Sun Yat-sen University, Shenzhen, Guangdong Province China; 3 Department of Obstetrics and Gynecology, Subei People’s Hospital, Yangzhou, Jiangsu Province China; 4 Medical College of Yangzhou University, Yangzhou, Jiangsu Province China; 5 Ningbo Center for Disease Control and Prevention, Ningbo, China

**Keywords:** Maternal near-miss, Maternal mortality, Maternal morbidity, Intensive care unit

## Abstract

**Background:**

Analysis of “maternal near-misses” is expected to facilitate assessment of the quality of maternity care in health facilities**.** Therefore, this study aimed to investigate incidence, risk factors and causes of maternal near-misses (MNM) admitted to the intensive care unit (ICU) within five years by using the World Health Organization’s MNM approach.

**Methods:**

A five-year retrospective study was conducted in Subei People’s Hospital of Yangzhou, Jiangsu Province from January 1, 2015 to December 31, 2019. Risk factors in 65 women with MNM in the intensive care unit (ICU) were explored by using chi-square tests and multivariable logistic regression analysis. Causes and interventions in MNM were investigated by descriptive analysis.

**Results:**

Average maternal near-miss incidence ratio (MNMIR) for ICU admission was 3.5 per 1000 live births. Average maternal mortality ratio (MMR) was 5 per 100,000 live births. MI for all MNM was 0.7%. Steady growth of MNMIR in ICU was witnessed in the five-year study period. Women who were referred from other hospitals (aOR 3.32; 95%CI 1.40–7.32) and had cesarean birth (aOR 4.96; 95%CI 1.66–14.86) were more likely to be admitted in ICU. Neonates born to women with MNM admitted in ICU had lower birthweight (aOR 5.41; 95%CI 2.53–11.58) and Apgar score at 5 min (aOR 6.39; 95%CI 2.20–18.55) compared with women with MNM outside ICU. ICU admission because of MNM occurred mostly postpartum (*n* = 63; 96.9%). Leading direct obstetric causes of MNM admitted in ICU were hypertensive diseases of pregnancy (*n* = 24; 36.9%), followed by postpartum hemorrhage (*n* = 14; 21.5%), while the leading indirect obstetric cause was heart diseases (*n* = 3; 4.6%).

**Conclusions:**

Risk factors that were associated with MNM in ICU were referral and cesarean birth. Hypertensive disease of pregnancy and postpartum hemorrhage were the main obstetric causes of MNM in ICU. These findings would provide guidance to improve professional skills of primary health care providers and encourage vaginal birth in the absence of medical indications for cesarean birth.

## Background

Reducing maternal mortality is a primary issue of global concern. The global maternal mortality ratio (MMR) was reduced to 216 per 100,000 live births in 2015 [1]. According to the Sustainable Development Goals (SDG), global MMR should be reduced to less than 70 per 100,000 live births and no individual country should have MMRs > 140 per 100,000 live births in 2030 [1–3]. MMR is one of the vital indicators to measure socio-economic status, resource allocation as well as quality of maternal and child health care in a country or region [4].

With modern medical technology, maternal deaths have become rare, especially in high income countries. Because of lower deaths nowadays, it cannot monitor and evaluate the quality of obstetric care accurately [5]. In view of this, the World Health Organisation (WHO) introduced the concept of maternal near-miss (WHO-MNM) in 2009, which clarifies the diagnostic criteria from three aspects: clinical standards, laboratory tests and disease management standards. WHO-MNM is defined as a pregnant woman who was on the verge of death during pregnancy, childbirth and postpartum within 42 days, but was successfully rescued and continued to survive due to good management or luck [6]. MNM has similar characteristics and pathophysiological processes as maternal deaths [7]. While some women with severe acute complications die during pregnancy, childbirth or puerperium, a proportion of them narrowly escapes death. Analysis of “MNM” and maternal deaths is expected to facilitate assessment of the quality of obstetric care in health facilities and accessibility to reliable and objective data to reduce severe maternal morbidity and prevent maternal death. A global unified diagnostic approach is more conducive to guidance, tracking and policymaking [8, 9].

China achieved the Millennium Development Goals in 2015 [10]. The national strategy ‘Healthy China 2030’ states that China’s MMR should be reduced to 12 per 100,000 live births in 2030 [11]. In 2016, “the universal two-child policy” that one couple can have two children, started to be implemented, replacing the earlier “one child policy”. Demographic structural changes will not only increase the number of older women, but also heighten the incidence and risks of complications and comorbidities of pregnancy and childbirth [12, 13]. Joint management of women with MNM by obstetricians and ICU-specialists is a major measure for the rescue of emergency obstetric care. Incidence of MNM in ICU can be used as an important indicator to judge life-threatening obstetric complications [14]. A multicountry survey by WHO showed that use of high-quality ICU is notably correlated with decline in MMR [15]. As a result, it is necessary to predict, identify and manage MNM admitted to ICU to improve risk prevention and control systems.

As one of the emergency centers for MNM in Yangzhou, our hospital has abundant experience in joint management of women with obstetric complications admitted to ICU. So far, there is no report on monitoring MNM in Yangzhou. Therefore, our aim is to explore incidence, risk factors and causes of MNM admitted to ICU from 2015 to 2019 in order to provide clinical experience and lessons for reducing adverse maternal outcomes and standardise the process of maternal health care.

## Method

This study was conducted in Subei People’s Hospital (SPH) of Yangzhou, Jiangsu Province from January 1, 2015 to December 31, 2019. As a tertiary general hospital, SPH integrates clinical, teaching and scientific research in the field of obstetrics. The obstetric department is equipped with 116 beds, 95 medical staff, and advanced medical equipment such as integrated obstetric beds, an obstetric central monitoring system, Doppler fetal heart rate monitor, etc. In 2016, it became one of the Critical Maternal Emergency Centers for MNM in Yangzhou, with an emergency green channel for midwifery agencies in the region and established a multidisciplinary expert team to provide 24-h emergency obstetric care.

All women who met any of the clinical, laboratory or management standards set by WHO at any trimester of pregnancy or within 42 days after birth were included. All women with MNM were divided into those admitted to ICU (ICU-group) and those with MNM who were not admitted to ICU (non-ICU group). Meanwhile, a standardised data collection mechanism in conjunction with the head of department and the head nurse was developed. Prior to data collection, two researchers were provided with unified training, which included the purpose of study, WHO-MNM approach, use of standardised forms, data collection process, relevant indicators in the form and definitions of complications.

For every woman with MNM, contents of general demographic and obstetric data include age, citizenship, region, referral, parity, gestational age, prenatal examinations, previous cesarean birth, mode of birth, method of conception, hospitalisation time, timing in ICU admission, length of stay in ICU, neonatal sex, birthweight, Apgar score (1 min, 5 min) and neonatal outcome.

Medical diagnosis was coded under the International Classification of Diseases (ICD-10). When several complications coexisted, only the major cause of admission to ICU was considered. They were divided into direct and indirect obstetric causes. Direct obstetric causes included hypertensive disorders of pregnancy, abruptio placenta, ectopic pregnancy, intrahepatic cholestasis of pregnancy, acute fatty liver of pregnancy, postpartum hemorrhage, amniotic fluid embolism, ruptured uterus, severe obstetric infection and others. Indirect obstetric causes included heart diseases, lung infection, gastrointestinal disorders, hematological diseases, neurologic diseases, mental diseases, acute pancreatitis and renal diseases.

Whether admitted to ICU or not, MNM clinical interventions in our hospital included blood transfusion (≥5 units red blood cells), hysterectomy, uterine compression sutures, radiological arterial embolization and arterial balloon surgery. However, ICU-specific interventions included continuous use of drugs, noninvasive assisted ventilation, invasive blood pressure monitoring, plasmapheresis, renal dialysis, endotracheal intubation and deep/central venous catheterization.

If there was any doubt about missing data, the attending doctor was consulted. Through obstetric birth records, annual births data were obtained. Data collection period was from June 1, 2020 to July 31, 2020. Main researchers and experts regularly checked completeness and consistency of data and proposed amendments and improvements on the spot.

### Outcome indicators

- Maternal near-miss incidence ratio (MNMIR): the number of women with MNM per 1000 live births.
- Maternal mortality ratio (MMR): the number of maternal deaths per 100,000 live births.
- Mortality index (MI) = numbers of maternal deaths per total numbers of maternal deaths and MNM.

### Statistical analysis

Data were entered and organised by Microsoft Excel 2007 and analysis was performed by IBM SPSS statistics data editor version 26.0. Descriptive analysis was expressed in frequency, percentage and means with standard deviations. Cross tables were used to explain associations between variables and adverse outcomes. Chi-Square tests were used to compare proportions and derived potential risk factors for ICU admission. All variables with *p* < 0.15 in univariate analysis were included in the multivariate model, which used adjusted OR (aOR) and 95% confidence intervals (95% CI) to assess the impact of risk factors. Statistical significance was considered at *p*-value < 0.05.

## Result

**Fig. 1:**
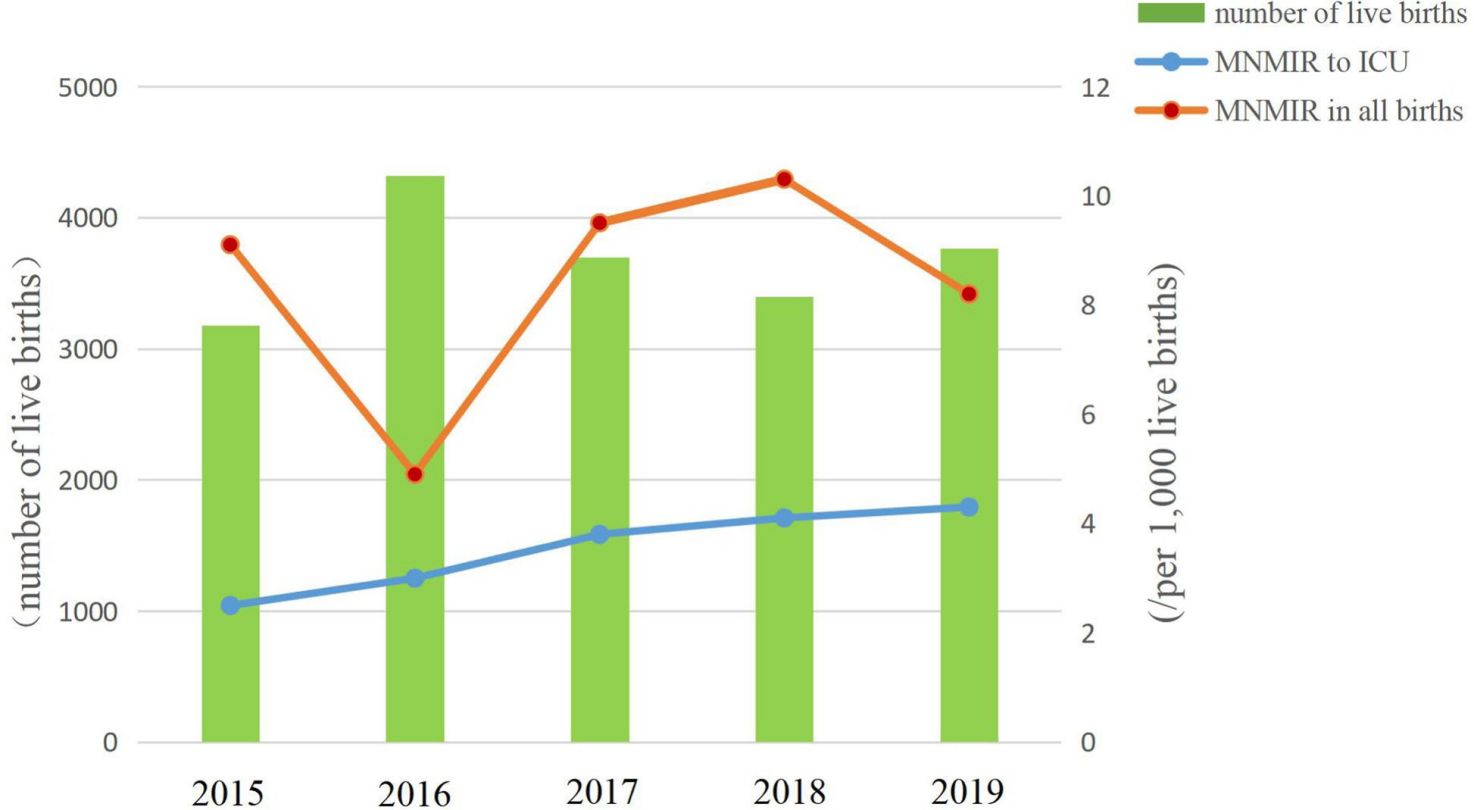
Change trend of birth volume and MNMIR* admission to ICU from 2015 to 2019. *MNMIR is defined as the number of maternal near miss cases per 1000 live births

Over the 5 years of the study (2015–2019), information from 17,843 women in SPH was enrolled. There were 18,357 live births, including 17,330 singletons, 512 twins and 1 triplets. One maternal death occurred in 2016 from acute heart failure due to severe preeclampsia, irregular prenatal examinations and not taking her medication as prescribed. Average MMR in SPH was 5 per 100,000 live births. MI for all MNM was 0.7%. A total of 151 women met the criteria of the WHO-MNM tool. Table 1 shows that average MNMIR in all births was 8.2 per 1000 live births (Table 1). As a result of the “ universal two-child policy “, it is clear that the MNMIR in all births fell to its lowest point in 2016 due to a boom in births, rose to its peak in 2017 and then slowly declined (Fig. 1). The number of women admitted to ICU during pregnancy or after birth was 65. Average MNMIR for admission to ICU was 3.5 per 1000 live births. It increased from 2.5 per 1000 live births in 2015 to 4.3 in 2019. While average MNMIR outside ICU was 4.7 per 1000 live births. It reached a bottom to 1.9 per 1000 live births in 2016, then reached the top to 6.1 per 1000 live births in 2018 and dropped to 3.9 per 1000 live births in 2019.

**Table 1:** Maternal statistics cohort 2015 to 2019

Year	Total number of live births	MNM Per 1000 live births. N (‰)	MNM in ICU^a^ Per 1000 live births N (‰)
2015	3179	29(9.1)	8(2.5)
2016	4319	21(4.9)	13(3.0)
2017	3698	35(9.5)	14(3.8)
2018	3398	35(10.3)	14(4.1)
2019	3763	31(8.2)	16(4.3)
total	18,357	151(8.2)	65(3.5)

Abbreviations:*MNM* maternal near miss; *ICU* intensive care unit

^a^MNM in ICU is defined as maternal near miss admitted to the ICU

Mean age of MNM in ICU was 34.5 ± 4.77 and 30.1 ± 5.13 years outside ICU. Average gestational age in ICU was 34 ± 3.9 weeks (range 21–40 weeks) and 34 ± 5.0 (range14–41 weeks) outside ICU. Mean age and gestational age were, however, not statistically significantly different between the two groups in the univariate analysis. Other socio-demographic characteristics of women were summarized in Table 2. Compared with MNM outside ICU, MNM in ICU were more likely to be referred (aOR 3.32; 95%CI: 1.40–7.32) and gave birth by cesarean (aOR 4.96; 95%CI 1.66–14.86). The percentage of cesarean birth in ICU (92.7%) was significantly higher than the overall 5-year cesarean birth rate in the hospital (46.1%). Most women with MNM (*n* = 63; 96.9%) were admitted in ICU postpartum. Average length of ICU stay was 3.7 ± 2.3 days.

**Table 2:** Demographic and obstetrical characteristics of women with maternal near-miss in two groups

Characteristics	ICU group		None-ICU group			
	N	%	N	%	OR 95%CI	^b^aOR 95%CI
Age						
< 35	51	78.5	69	80.2	1 (ref.)	
≥ 35	14	21.5	17	19.8	0.90(0.41,1.99)	
Citizenship						
Local	49	75.4	66	76.7	1 (ref.)	
Migrant	16	24.6	20	23.3	0.93(0.44,1.97)	
Region						
Rural	42	64.6	41	47.7	**0.50(0.26,0.97)**	1.67(0.79,3.52)
Urban	23	35.4	45	52.3	1 (ref.)	1 (ref.)
Gestational age						
< 37	52	80.0	60	69.8	0.58(0.27, 1.24)	
≥ 37	13	20.0	26	30.2	1 (ref.)	
Referral						
No	38	58.5	69	80.2	1 (ref.)	1 (ref.)
Yes	27	41.5	17	19.8	**0.35(0.17,0.72)**	**3.32(1.40, 7.32)**
Parity						
P0	7	10.8	5	5.8	1 (ref.)	1 (ref.)
P1	18	27.7	39	45.3	3.03(0.85, 10.87)	0.26(0.07, 1.03)
*P* ≥ 2	40	61.5	42	48.9	1.14 (0.43, 5.00)	0.73 (0.20, 2.70)
Prenatal examinations						
Regular	51	78.5	75	87.2	1 (ref.)	
Irregular	14	21.5	11	12.8	0.53(0.22,1.27)	
Previous cesarean birth						
0	41	63.1	56	65.1	1 (ref.)	
≥ 1	24	36.9	30	34.9	0.91(0.47,1.79)	
Mode of birth						
Cesarean birth	60	92.3	63	73.3	**0.23(0.08, 0.64)**	**4.96(1.66, 14.86)**
Vaginal birth	5	7.7	23	26.7	1 (ref.)	1 (ref.)
Method of conception						
Spontaneous	58	89.2	75	87.2	1 (ref.)	
ART ^a^	7	10.8	11	12.8	1.22(0.44, 3.33)	
Hospitalization time						
≤ 7	15	23.1	22	25.6	1 (ref.)	
> 7	50	76.9	64	74.4	0.87(0.41, 1.86)	
Timing in ICU admission						
Antepartum	2	3.1				
Postpartum	63	96.9				
Length of stay in the ICU						
1–3	35	53.8				
≥ 4	30	46.2				

^a^ ART is defined as assisted reproductive technology

^b^OR adjusted by region, referral, parity and mode of delivery

*Ref* reference category

Bold value refers: statistically significant association

Regarding neonatal characteristics, there was a significant difference in low birthweight (aOR 5.41; 95%CI: 2.53–11.58) and low Apgar scores at 5 min (aOR 6.39; 95%CI: 2.20–18.55) (Table 3).

**Table 3:** Neonatal characteristics of maternal near-miss in two groups

Neonatal characteristics	ICU group		None-ICU group			
	N	%	N	%	OR 95%CI	^a^aOR 95%CI
All live newborns	64	–	90	–		
Neonatal sex						
Male	30	46.9	41	45.6	0.95 (0.50,1.80)	
Female	34	53.1	49	54.4	1 (ref.)	
Number of Neonates						
Singletons	52	89.7	72	88.9	0.92 (0.31, 2.75)	
Twins	6	10.3	9	11.1	1 (ref.)	
Birthweight						
≥ 2500	16	25.0	57	63.3	1 (ref.)	1 (ref.)
< 2500	48	75.0	33	36.7	**0.19(0.09, 0.39)**	**5.41 (2.53, 11.58)**
Apgar score at 1 min						
≥ 7	42	65.6	66	73.3	1 (ref.)	
< 7	22	34.4	24	26.7	0.69 (0.35, 1.39)	
Apgar score at 5 min						
< 7	20	31.3	6	6.7	**0.16 (0.06, 0.42)**	**6.39 (2.20, 18.55)**
≥ 7	44	68.7	84	93.3	1 (ref.)	1 (ref.)
Admission to Neonatal Department						
Yes	37	57.8	41	45.6	1.64 (0.86,3.13)	1.72 (0.83, 3.60)
No	27	42.2	49	54.4	1 (ref.)	1 (ref.)

^a^OR adjusted by birthweight, Apgar score at 5 min and admission to Neonatal Department

*Ref* reference category

Bold value refers: statistically significant association

Overall, direct obstetric causes were the primary reasons for MNM (129/151; 85.4%). Leading direct obstetric causes of MNM in ICU were hypertensive disorders of pregnancy (*n* = 24; 36.9%) and postpartum hemorrhage (*n* = 14; 21.5%). The leading indirect obstetric cause of MNM admitted to the ICU was heart diseases (*n* = 3; 4.6%). Outside ICU the most common direct obstetric cause was postpartum hemorrhage (*n* = 67; 77.9%), followed by hypertensive disorders of pregnancy (*n* = 5; 5.8%). Neurologic diseases (*n* = 8; 9.2%) were the leading indirect obstetric cause. Major cause of postpartum hemorrhage in both groups was both placenta accrete/placenta previa (*n* = 7;10.8% in ICU and *n* = 40;46.4% outside ICU) (Table 4).

**Table 4:** Causes of maternal near-miss in two groups

Cause^*^	MNM	ICU group		None-ICU group	
	N	N	%	N	%
**Direct obstetric causes**	129	53	81.5	76	88.4
**Hypertensive disorders of pregnancy**	29	24	36.9	5	5.8
HELLP syndrome	2	2	3.1	0	0
Eclampsia	12	8	12.3	4	4.6
Chronic hypertension with preeclampsia	5	5	7.7	0	0
Severe preeclampsia	10	9	13.8	1	1.2
**Abruptio placenta**	5	4	6.2	1	1.2
**Ectopic pregnancy**	1	0	0	1	1.2
**Intrahepatic cholestasis of pregnancy**	1	1	1.5	0	0
**Acute fatty liver of pregnancy**	3	3	4.6	0	0
**Postpartum hemorrhage**	81	14	21.5	67	77.9
Uterine atony	31	6	9.2	25	29.1
Placenta accreta/ Placenta previa	47	7	10.8	40	46.4
Coagulation disorders	2	1	1.5	1	1.2
Soft birth canal injury	1	0	0	1	1.2
**Amniotic fluid embolism**	3	3	4.6	0	0
**Ruptured uterus**	3	1	1.5	2	2.3
**Severe obstetric infection**	1	1	1.5	0	0
**Others**	2	2	3.2	0	0
**Indirect obstetric causes**	22	12	18.5	10	11.6
Heart diseases	4	3	4.6	1	1.2
Lung infection	1	1	1.5	0	0
Gastrointestinal disorders	1	1	1.5	0	0
Hematological diseases	3	2	3.2	1	1.2
Neurologic diseases	9	1	1.5	8	9.2
Mental diseases	1	1	1.5	0	0
Acute pancreatitis	2	2	3.2	0	0
Renal diseases	1	1	1.5	0	0

Whether admitted to the ICU or not, MNM interventions included blood transfusion(≥5 units red blood cells), hysterectomy, uterine compression sutures, radiological arterial embolization and arterial balloon surgery (Table 5). Among the women admitted to the ICU, 15 received continuous administration of vasoactive drugs (dopamine/norepinephrine/epinephrine). Two women needed plasmapheresis, two renal dialysis, 24 endotracheal intubation and 34 noninvasive assisted ventilation. Fourteen women were given continuous invasive blood pressure monitoring and because of large fluctuations of blood pressure and hypertensive crisis, 18 women had blood pressure monitoring by deep venipuncture.

**Table 5:** Clinical interventions of MNM in and outside ICU

Clinical interventions	ICU group		None-ICU group	
	*N* = 65	%	*N* = 86	%
Blood transfusion (≥5 units red blood cells)	22	33.8	67	77.9
Continuous use of vasoactive drugs	15	23.1		
Noninvasive assisted ventilation	34	52.3		
Invasive blood pressure monitoring	14	21.5		
Plasmapheresis	2	3.1		
Hysterectomy	9	13.8	5	5.8
Uterine compression sutures	4	6.2	18	20.9
Radiological arterial embolization	5	7.7	23	26.7
Renal dialysis	2	3.1		
Endotracheal intubation	24	36.9		
Deep/Central venous catheterization	18	26.7		
Arterial Balloon Surgery	1	1.54	4	4.7

## Discussion

In this study, average MNMIR for ICU admission was 3.5 per 1000 live births, using WHO-MNM criteria. Our MNMIR was higher than that in high income countries, such as New Zealand (2.1) and Portugal (0.7) [16, 17]. An explanation could be that our study was in a referral tertiary hospital with a high migrant population.

In China, secondary and tertiary hospitals with strong obstetric strength and comprehensive treatment capabilities need to set up ICU in order to guarantee treatment beds for MNM. ICU-MNM is lower than for all women with MNM [18]. In this study, most women with MNM were admitted to ICU after birth. Average length of stay in ICU was 3.7 ± 2.3, which is higher compared to another study in Iran [19]. Our tertiary general hospital was able to recognise the importance of ICU care and provided adequate numbers of beds for MNM.

We found statistically significant differences between the characteristics of the ICU group compared to those outside ICU in terms of region, referral and mode of birth in agreement with studies from rural Rwanda [20], Italy [21] and WHO’s 2019 multicountry survey on maternal and newborn health [22]. Referral from another health facility was a significant risk factor associated with ICU admission as showed in another study in Canada [23]. This suggests a strong need to improve training of primary health care providers and their ability to care for complex situations, making timely and appropriate referrals. Effective regional cooperation between doctors at lower levels of care should also be strengthened.

Cesarean birth was associated with ICU admission, similar to a study from Italy [24]. Cesarean birth may increase risks of bleeding and sepsis, leading to hysterectomy and longer hospital stays, but can also be a protective factor against adverse outcomes [25, 26]. We did not find an association between previous cesarean birth and ICU admission, in contrast with the WHO’s multicountry study [22]. Unfortunately, vaginal birth after cesarean birth is not yet practised in Yangzhou. Previous cesarean birth rate was 33.9% in this study, unfortunately leading to at least 33.9% repeat cesareans [14, 27]. The number of scarred uteri in China doubled from 2012 (9.8%) to 2016 (17.7%) [28]. The cesarean birth rate in China was the highest among the nine Asian countries, especially because of repeat cesareans and those without medical indications [22].

Main obstetric causes of MNM in ICU were hypertensive disorders of pregnancy (36.9%) and postpartum hemorrhage, in line with studies in Finland [29] and low-income countries [30, 31]. In pregnancy 5–10% of women will develop hypertensive disorders, affected by race, environment and socio-economic status [32]. Irregular prenatal examinations and low educational attainment were risk factors for hypertensive disorders of pregnancy [33]. Women with severe preeclampsia, eclampsia or HELLP syndrome should be hospitalised in time, treated with magnesium sulfate, antihypertensive drugs and corticosteroids to enhance fetal lung maturation. Evidence shows that planned birth can reduce maternal morbidity and development of severe hypertensive disorders (especially systolic hypertension), shorten hospital stay and lower treatment costs, although preterm birth can lead to increased neonatal hospitalization [32].

Postpartum hemorrhage tends to easily have serious adverse effects on women’s physical and mental health. Placenta accreta or previa was the most common cause in the study. Our high rates of cesarean birth led to placenta accreta and because of referral from other hospitals our data will be biased. Blood transfusion is an important emergency intervention and criteria for blood transfusion in MNM have not changed. We actively encourage autologous blood transfusion to ensure scientific rationality of clinical blood use, avoid blood waste and reduce blood shortages. The inclusion criterium of ≥5 units of red blood cells may not truly reflect the severity of MNM, because availability of blood and the threshold of use is different in various regions [34]. Higher rates of hysterectomy are related to delay in seeking medical care and referrals [35, 36]. In recent years, however, a reasonable choice of arterial balloon catheter placement, compression sutures and radiological artery embolization has effectively reduced hysterectomy rates and improved maternal quality of life [37].

Although direct obstetric causes are the main reasons for MNM in ICU, obstetric complications and comorbidities tend to increase in women with underlying diseases, making management of indirect obstetric causes complex and sometimes beyond the capacity of obstetricians [38]. The main indirect obstetric cause of MNM in ICU was heart diseases, while neurologic diseases, especially epilepsy, were the most frequent outside ICU. MMR caused by heart-diseases rose in China from 1.8 in 2013 to 3.3 per 100,000 live births in 2016 [39]. Our tertiary hospital provided comprehensive management and advanced interventions for women suffering from those diseases and used multidisciplinary management to reduce MMR significantly.

Women in ICU were managed with continuous use of vasoactive drugs, plasmapheresis, renal dialysis, ventilator-assisted respiration, continuous invasive blood pressure monitoring, non-invasive assisted ventilation and implantation of temporary pacemakers. Women with MNM outside ICU most often had severe postpartum hemorrhage caused by placenta previa or accrete and received more treatment of massive blood products infusion, arterial embolization and arterial balloon implantation.

Only one maternal death occurred in our setting from severe preeclampsia. MMR was 5 per 100,000 per 100,000 livebirths, indicating a MI of 0.7% for all births. Compared with low and low-middle-income countries, this reflects our hospital’s good health care and the availability of high-quality drugs and interventions [4]. Regular audit and analysis of MNM in ICU is a valuable method for health care personnel to learn lessons from what happened during their work.

Neonates from mothers in ICU had higher neonatal intubation rates, NICU transfer rates and lower Apgar scores [40]. This shows that ICU health care personnel are facing huge challenges. They need to pay attention not only to the physical condition of women with MNM, but also to the condition of newborns to provide timely treatment.

Systematic investigation of the risk factors that result in MNM admitted to the ICU, may lead to useful information to reduce serious maternal morbidity and provide references for policymakers [41].

Improving the quality of obstetric care to reduce maternal morbidity and mortality has attracted global attention [42]. Since 2017, maternity management in China has been marked by five colors (green, yellow, orange, red and purple) according to the severity of risks. When changes in maternal health are detected, medical institutions should immediately conduct dynamic pregnancy risk assessments and adjust pregnancy risk classifications [43].

### Strengths and limitations

This study is the first retrospective study of MNM in ICU in Yangzhou. Obstetricians in this study have abundant experience in handling pregnancy complications and they are highly aware of the incidence, risk factors, and causes of MNM, which is helpful to promote the transformation from quantity to quality of obstetric health. Moreover, the five-year study can objectively evaluate the impact of the “two-child policy” on MNM.

There are some limitations of our study. It was conducted in a single tertiary hospital and thus our results may not be generalized to primary and private hospitals. However, most women with MNM receive treatment in tertiary general hospitals in China. Secondly, this is a retrospective study with data from medical records. It is hoped that data loss can be minimised with the exploitation and development of tools for automatic identification of MNM [34]. Moreover, small sample size tended to result in wide 95%Cl and thus in poorer precision to estimate impact factors. In a future study, study period could be extended or hospitals added to improve precision. Finally, our use of WHO-MNM criteria may underestimate the number of severe maternal morbidities [9]. Further research is needed to establish MNM standards suitable for our country or region [44–47].

## Conclusion

Average MNMIR in ICU was 3.5 per 1000 live births, increasing to 8.2 per 1000 LBs in all births. Risk factors associated with MNM in ICU were referral and cesarean birth. Hypertensive disorders of pregnancy and postpartum hemorrhage were the main obstetric causes of MNM in ICU. These findings would guide to improve professional skills of primary health care providers and encourage vaginal birth in the absence of medical indications for cesarean birth. Regular monitoring and audit of MNM could help improve the quality of maternity care.

## Data Availability

Our data came from the case system of Subei People’s Hospital. The data in this research report is available for use with the permission of the corresponding author.

## Abbreviations

ICU: Intensive care unit
MMR: Maternal mortality ratio
MI: Mortality index
MNMIR: Maternal near-miss incidence ratio
MNM: Maternal near miss
WHO: World Health Organization
ART: Assisted reproductive technology
