# Correction to: Women with maternal near-miss in the intensive care unit in Yangzhou, China: a 5-year retrospective study

**DOI:** 10.1186/s12884-021-04312-4

**Published:** 2021-12-27

**Authors:** Ying Chen, Jiaoyang Shi, Yuting Zhu, Xiang Kong, Yang Lu, Yanru Chu, Miskatul Mustafa Mishu

**Affiliations:** 1 School of Nursing, Yangzhou University, Yangzhou, Jiangsu Province China; 2 Department of Obstetrics, The Eighth Affiliated Hospital of Sun Yat-sen University, Shenzhen, Guangdong Province China; 3 Department of Obstetrics and Gynecology, Medical College of Yangzhou University, Yangzhou, Jiangsu Province China; 4 Medical College of Yangzhou University, Yangzhou, Jiangsu Province China; 5 Ningbo Center for Disease Control and Prevention, Ningbo, China

**Correction to: BMC Pregnancy Childbirth 21, 784 (2021)**

**https://doi.org/10.1186/s12884-021-04237-y**

Following publication of the original article [1], the authors identified an error in the affiliation of Xiang Kong.

The incorrect affiliation is: Department of Obstetrics and Gynecology, Subei People’s Hospital, Yangzhou, Jiangsu Province, China.

The correct affiliation is: Department of Obstetrics and Gynecology, Medical College of Yangzhou University, Yangzhou, Jiangsu Province, China.

The original article has been corrected.
